# A new cognitive evaluation battery for Down syndrome and its relevance for clinical trials

**DOI:** 10.3389/fpsyg.2015.00708

**Published:** 2015-06-04

**Authors:** Susana de Sola, Rafael de la Torre, Gonzalo Sánchez-Benavides, Bessy Benejam, Aida Cuenca-Royo, Laura del Hoyo, Joan Rodríguez, Silvina Catuara-Solarz, Judit Sanchez-Gutierrez, Ivan Dueñas-Espin, Gimena Hernandez, Jordi Peña-Casanova, Klaus Langohr, Sebastia Videla, Henry Blehaut, Magi Farre, Mara Dierssen, The TESDAD Study Group, Aida Cuenca-Royo

**Affiliations:** ^1^Human Pharmacology and Clinical Neurosciences Research Group-Neurosciences Program, IMIM-Hospital del Mar Medical Research InstituteBarcelona, Spain; ^2^Cellular and Systems Neurobiology Research Group, Systems Biology Program, Centre for Genomic RegulationBarcelona, Spain; ^3^Biomedical Research Centre in Physiopathology of Obesity and Nutrition (CIBEROBN)Santiago de Compostela, Spain; ^4^CEXS, Universitat Pompeu FabraBarcelona, Spain; ^5^Neurofunctionality of Brain and Language Group-Neurosciences Program, IMIM-Hospital del Mar Medical Research InstituteBarcelona, Spain; ^6^Fundació Catalana Síndrome de DownBarcelona, Spain; ^7^Drug Abuse Epidemiology Research Group-Epidemiology and Public Health Program, IMIM-Hospital del Mar Medical Research InstituteBarcelona, Spain; ^8^Fundació Privada Espai SalutBarcelona, Spain; ^9^Universitat Autónoma de BarcelonaUDIMAS, Barcelona, Spain; ^10^Department of Statistics and Operations Research, Universitat Politècnica de CatalunyaBarcelona, Spain; ^11^Fondation Jérôme LejeuneParis, France; ^12^Universitat Autònoma de Barcelona, i Hospital Universitari Germans Trias i Pujol (IGTP)Barcelona, Spain; ^13^Biomedical Research Centre on Rare Diseases (CIBERER)Valencia, Barcelona, Spain

**Keywords:** Down syndrome, TESDAD neurocognitive battery, intellectual disabilities, cognition, clinical trials as topic

## Abstract

The recent prospect of pharmaceutical interventions for cognitive impairment of Down syndrome (DS) has boosted a number of clinical trials in this population. However, running the trials has raised some methodological challenges and questioned the prevailing methodology used to evaluate cognitive functioning of DS individuals. This is usually achieved by comparing DS individuals to matched healthy controls of the same mental age. We propose a new tool, the TESDAD Battery that uses comparison with age-matched typically developed adults. This is an advantageous method for probing the clinical efficacy of DS therapies, allowing the interpretation and prediction of functional outcomes in clinical trials. In our DS population the TESDAD battery permitted a quantitative assessment of cognitive defects, which indicated language dysfunction and deficits in executive function, as the most important contributors to other cognitive and adaptive behavior outcomes as predictors of functional change in DS. Concretely, auditory comprehension and functional academics showed the highest potential as end-point measures of therapeutic intervention for clinical trials: the former as a cognitive key target for therapeutic intervention, and the latter as a primary functional outcome measure of clinical efficacy. Our results also emphasize the need to explore the modulating effects of IQ, gender and age on cognitive enhancing treatments. Noticeably, women performed significantly better than men of the same age and IQ in most cognitive tests, with the most consistent differences occurring in memory and executive functioning and negative trends rarely emerged on quality of life linked to the effect of age after adjusting for IQ and gender. In sum, the TESDAD battery is a useful neurocognitive tool for probing the clinical efficacy of experimental therapies in interventional studies in the DS population suggesting that age-matched controls are advantageous for determining normalization of DS.

## Introduction

Down Syndrome (DS) is the most common genetic cause of mental retardation (Megarbane et al., 2009), with an incidence of approximately 9.65 for every 10,000 live births in Europe (Khoshnood et al., 2011). Although most of its phenotypic features are variable, both in prevalence and expression, the DS neurocognitive profile is characterized by psychomotor delay and a general, and pronounced, deficit in learning/memory, executive functions, and language abilities that shape the intellectual disability of the syndrome (Pennington et al., 2003; Rowe et al., 2006; Vicari, 2006; Iacono et al., 2010). The recent flourishing of therapy-oriented research in DS has led to an increasing number of clinical trials that require validated test batteries to test treatment efficacy and safety. Research in the field of cognitive enhancers for mental health is moving toward considering key brain networks and specific areas underlying major cognitive deficits as the main targets for therapeutic intervention, instead of focusing on broad-based neurotransmitter systems. In parallel, there is the pressing need to update the diagnostic classification schemes and the neuropsychological assessment methods according to this new neuroscience-based approach (Insel et al., 2010, 2013). Furthermore, few of the plethora of methods for cognitive assessment report clinically significant psychometric data for DS subjects, and neither do they suitably accommodate the heterogeneous range of impairments experienced by this population.

The prevailing methodology used to characterize cognitive functioning compares DS subjects, or those with other learning disabilities of genetic origin (e.g., Williams-Beuren and Fragile-X Syndromes) or unknown etiology, to healthy controls of the same “mental age.” The comparison is assumed to provide an index of global level of mental maturation (Edgin et al., 2010b; Finestack and Abbeduto, 2010; Costanzo et al., 2013a). These approaches are based on the notion that the mental maturation rate in subjects with intellectual disability differs substantially from typically developed subjects of equal chronological age, but should not differ significantly, or only in certain capacities, when matched for their “mental age” (Costanzo et al., 2013b). Whilst this perspective has been valuable for characterizing the DS cognitive phenotype, it is not useful for determining the gap in cognitive performance between DS subjects and healthy adults, which is the cognitive target we aim for in clinical trials. The few studies that have used an age-matched healthy population with standard norms have focused on the study of specific cognitive domains (e.g., language and memory processing), but have not carried out a comprehensive description of the DS profile (Næss et al., 2011).

Cognitive-enhancing therapies aim to bring cognitive and functional competence in DS closer to the standards expected for their chronological age. We propose, therefore, to use an age-matched healthy control population for the systematic evaluation of the reduction, stabilization, or slowing of the cognitive and functional performance of DS with respect to therapeutic interventions. From a clinical point of view, standard norms from healthy subjects provide a feasible and valuable reference for quantifying the magnitude of cognitive improvement needed for functional changes. For example, modest cognitive gains related to experimental treatments in DS subjects, which would be considered of subclinical magnitude in typically developed adults, could imply a mild but clinically meaningful and significant impact on everyday life functioning in the DS population, which is harder to determine using mental age or mentally disabled-matched subjects as a comparison.

We have developed the TESDAD battery for clinical trials to characterize the cognitive functioning of young adults with DS, within mild to moderate–severe intellectual disability, using standard norms from age-matched typically-developed adults as a reference for this characterization. The TESDAD battery was used to explore the relative contribution of intellectual quotient (IQ), gender, and age to neurocognitive variability among DS participants, and to identify specific relationships between cognitive performance and different aspects of functional outcome that could potentially serve for expecting functional change in interventional studies.

## Methods

### Participants

Eighty-six young adults of both genders with DS, aged 16–34 years, with any of the three DS genetic variations (trisomy 21, mosaic, or translocation) were enrolled in the study, mainly through the Fundació Catalana de Síndrome de Down (Barcelona) a local foundation specialized in providing health care services and educational programs to participants with DS and their families.

The data reported in the present work correspond to the baseline cognitive performance of a cohort of DS participants that participated in a clinical trial NCT01699711, that has been registered in https://clinicaltrials.gov/ct2/show/NCT01699711. All participants were drug free during the baseline assessment. Upon arrival at the research center (Hospital del Mar Medical Research Institute-IMIM), participants, parents and legal guardians (in case of legal incapacitation) were informed of the ensuing protocol and they gave their written informed consent before participating. Subjects with neurological disease other than DS, relevant medical disease, unstable co-morbid mental disorder or currently taking any treatment that could interfere with cognitive function were excluded from the study. Other exclusion criteria applied to all the participants were: (i) having suffered from any major illness or undergoing major surgery in the last 3 months before the study; (ii) new or irregular medication in the month preceding the study; (iii) current ingestion of vitamin or catechins supplements or Non-Steroidal Anti-Inflammatory Drugs (NSAIDs) in the 2 weeks preceding the study; (iv) history of gastrointestinal, hepatic, renal, or any other problems that may alter absorption, distribution, metabolism, or excretion of the drug. Genetic variations were documented by chromosomal analysis.

### Test procedure and customized neuropsychological test battery

The study was conducted in accordance with the ethical standards laid down in the Declaration of Helsinki and approved by the local ethics committee (CEIC-Parc de Salut Mar). At study onset the participants underwent medical examinations and a brief cognitive assessment to estimate their intellectual disability level based on criteria from the Diagnostic and Statistical Manual of Mental Disorders, 4th Edition-Text Revision (American Psychiatric Association, 1994). A trained evaluator then individually assessed the participants in a 90-min session aimed at exploring a wide range of cognitive and functional domains. The cognitive tests were presented in a fixed order to allow adequate intervals for delay trials on measures of episodic memory (see Supplementary Table B.1.). All tasks were carried out in a quiet, comfortable room. While participants completed the neuropsychological testing, parents, caregivers or legal guardians answered questionnaires measuring functionality in the participants' daily lives using questionnaires for the following domains: adaptive behavior, quality of life (QoL), quality of sleep, and neuropsychiatric symptoms. Measures of adaptive behavior were obtained with the adult version of the Adaptive Behavior Assessment System-Second Edition (ABAS-II). Quality of life was assessed with the parents'/guardians' version of the Kidscreen-27. Quality of sleep was explored with The Pittsburgh Sleep Quality Index (PSQI), and neuropsychiatric symptoms were assessed with the Neuropsychiatric Inventory (NPI). IQ was estimated using The Kaufman Brief Intelligence Test (K-BIT). A more detailed description of the complete neuropsychological battery and references can be found in the Supplemental Materials (A.1 and A.2). None of the participants required the presence of their parents or legal guardians to perform cognitive testing.

#### Neuropsychological testing

The following cognitive domains were explored: psychomotor speed, attention, episodic memory, executive functions, and language. Several tests from the Cambridge Neuropsychological Test Automated Battery (CANTAB) (Robbins and Sahakian, 1996) were employed in addition to standard paper and pencil tests. Psychomotor speed was measured with the Motor Screening Test (MOT, CANTAB). Attention was assessed by means of simple reaction time and span capacity measures using the Simple Reaction Time task (SRT, CANTAB), the Spatial Span forward recall (SSP, CANTAB) and the Digit Span forward recall from the Wechsler Adult Intelligence Scale-III (WAIS-III) that evaluated visual and verbal information, respectively. Measures of visual episodic memory and learning were obtained using the CANTAB Paired Associates Learning (PAL) and the Pattern Recognition Memory Test (PRM, CANTAB), and verbal episodic memory using the Cued Recall Test (CRT). Regarding executive functioning, fractioned components of verbal fluency, working memory, planning, mental flexibility, and inhibitory control were explored. Verbal word fluency was measured by means of the semantic fluency word generation task (animals in 1 min). Working memory for visual and verbal information was assessed with the Spatial Span backward recall (SSP, CANTAB) and the Digit Span backward recall (WAIS-III), respectively. Planning capacity was measured using the Tower of London from Drexel University (ToLDx) and mental flexibility with the Weigl Color-Form Sort Test. The Cats and Dogs Test was used to assess response inhibition. Finally, measures of expressive and receptive language were obtained by means of the Boston Naming Test and the Token Test, respectively. Only adult versions of the selected cognitive tests were employed with the exception of three specific tests for adults with intellectual disability due to the complexity of the tasks. These included the assessment of verbal episodic memory (Cued Recall Test), executive components of inhibition (Cats and Dogs) and mental flexibility (Weigl Sorting Test). We also administered the child's version of the ToLDx for the planning task to avoid floor effects. The cognitive tests were presented in a fixed order to allow adequate intervals for delay trials on measures of episodic memory. In addition, parallel versions of episodic memory tests were used to control for learning effects. Regarding the tests selected from the CANTAB, only clinical versions were administered.

To perform the comparison of our sample of DS participants with typically developed participants, test scores from normative data provided by the test publishers and normative studies for subjects of the same age range of our study (16–39 years) were employed. (1) For the analyses of CANTAB tests, we used norms derived from 51 to 199 control subjects reported in the CANTAB standard norm database (see Robbins et al., 1997, 1998, for a description of part of these data^1^. The Cambridge Cognition website http://www.cambridgecognition.com/technology provides a practical demonstration of the tests used). (2) For the analyses of paper and pencil tests, we used normative data from 84 to 87 participants (18–34 years old) from the Spanish Multicenter Normative Studies NEURONORMA young adults Project; (Peña-Casanova et al., 2012). (3) For the analyses of ToLDx results, as a child's version had been used, normative data from 76 participants (13–15 years) was selected (Culbertson and Zillmer, 2005), so that it better matched our sample. Similar analyses could not be carried out for performance on verbal episodic memory, mental flexibility, and response inhibition, due to the lack of normative data from typically developed adults for these tests.

This battery was developed, and is currently being used, in a longitudinal, randomized, double blind, placebo-controlled Phase II trial conducted by our research team in young adults with Down syndrome (the TESDAD study; De la Torre et al., 2014). In the present work, only baseline neurocognitive results from the TESDAD study are reported.

## Statistical analysis

The first step consisted of a descriptive analysis of the sociodemographic and clinical parameters of all the participants at baseline. Descriptive analyses were also carried out for all neuropsychological variables, providing measures of mean, standard deviation, and maximum and minimum values in the case of quantitative variables. In order to detect the presence of significant ceiling or floor effects in the variables, frequencies and percent were computed. Variables in which more than 10% of the sample obtained the maximum or the minimum score, and/or exhibited a significant absolute skewness index (>2) were categorized as having ceiling or floor effects.

We compared the DS group test scores of each cognitive variable to previously published normative data from age-matched healthy controls. Test scores of our DS participants could not be compared to normative data for those tests specifically developed for the assessment of participants with intellectual disability (CRT, Weigl, Cats and Dogs). In order to quantify and determine the gap between DS and normative groups, Cohen's effect size (“Cohen's *d”*), which is the difference of the means of two independent samples divided by the pooled standard deviation, together with its 95% confidence interval was calculated for all cognitive variables (Choen, 1988). Effect size differences higher than one and a half pooled standard deviations (|*d*| > 1.5) in cognitive performance between DS participants and age-matched normally developed adults were considered key cognitive processes substantially impaired in DS. In order to assess the severity of impairment the following categories were established: severe impairment (effect size differences larger than three pooled standard deviations: |*d*| > 3); substantial impairment (|*d*| > 1.5); moderate impairment (|*d*| > 1); and mild impairment (|*d*| > 0.5).

To study possible differences in cognitive and functional performance according to IQ, gender, and age, ANCOVA models were fitted for all neurocognitive measures including these three variables of interest. For the analyses, the IQ was categorized into two groups: mild/moderate (IQ ≥ 40) and severe (IQ <40) within the range of mental disability level. Concerning the two categorical variables, these models provide an adjusted estimation of the mean differences between persons with DS with IQ < 40 and persons with DS with IQ ≥ 40, on one hand, and female and male persons with DS, on the other hand. In case of variable age, the models provide an adjusted estimation of the mean difference associated to 1 year of age difference in persons with DS. The differences were considered to be statistically significant if the resulting *p*-value was less than 0.05. Finally, to explore the relationships between cognitive performance and functional outcome, the Pearson correlation coefficient was calculated to determine associations between cognitive variables, IQ (K-BIT standardized score) and functional outcomes of adaptive behavior and quality of life. We only report moderate and strong correlations (*r* ≥ 0.4). All statistical analyses were performed with the statistical software package R (The R Foundation for Statistical Computing), v3.0.2.

## Results

### Descriptive demographic and clinical data of the participants

Socio-demographic data and clinical parameters of the 86 DS participants are provided in Table 1. 51.2% were male and the mean age was 23.3 years [standard deviation (*SD*) = 4.3 years; range 16–34 years]. The median IQ for the full sample was 41 [K-BIT standardized score: 105 (*SD* = 17.8; range 80–180; IQ score (*SD*) = 8.3; range 40–86)], concentrating a slightly higher proportion of participants with moderate intellectual disability (IQ ≥ 40: 58.1%; *n* = 50) in comparison to those within the severe mental disability range (IQ < 40: 41.9%; *n* = 36). In terms of gender, the median IQ for males was 40 [K-BIT standardized score: 102 (*SD* = 19; range 80–180)] and 42 for females [K-BIT standardized score: 108 (*SD* = 16; range 80–154)]. The average years of schooling (regular school attendance in specialized or non-specialized educational centers) was 13 (*SD* = 1.9; range 10–18). In terms of DS genetic variations, the sample showed the usual proportion for this population: most participants had full trisomy 21 (simple: 95.3%, *n* = 82), two participants translocation (2.3%), one partial trisomy (1.1%), and one mosaic (1.1%).

**Table 1:** **Sociodemographic characteristics and clinical parameters at baseline**.

	**(*n* = 86)**
**AGE**	23.3 (4.3)
**GENDER**	
Female	42 (48.8%)
Male	44 (51.2%)
**EDUCATION (YEARS)^a^**	13 (1.9)
**HANDEDNESS**	
Right	67 (79.8%)
Left	17 (20.2%)
**INTELLECTUAL DISABILITY LEVEL**	
Mild/moderate (IQ ≥ 40)	50 (58.1%)
Severe (IQ < 40)	36 (41.9%)
**INTELLECTUAL QUOTIENT (IQ)**	
IQ	41^b^
K-BIT standardized score	105 (17.8)
Male (standardized; IQ)	102 (19); 40^b^
Female (standardized; IQ)	108 (16); 42^b^
**DS GENETIC VARIATIONS**	
Trisomy 21	82 (95.3%)
Mosaic	1 (1.1%)
Translocation	2 (2.3%)
Partial	1 (1.1%)

*Results are presented as mean (standard deviation) for continuous variables and absolute frequency (relative frequency) for categorical variables*.

a *Average years of school attendance in specialized or non-specialized educational centers*.

b *Only the median is reported because values below 40 cannot be determined exactly*.

From the eighty-six participants that participated in the study, 75 were able to reliably complete all cognitive procedures at baseline. Eleven participants could not perform the entire cognitive assessment protocol due to cognitive or behavioral alterations that interfered with testing. From those, 7 participants presented marked language deficit (significant speech and/or comprehension limitations), and 3 participants presented behavioral disturbances or mental block. One case showed poor collaboration during the assessment. Only data from these 11 participants for those tests successfully completed were included in the analyses.

Ceiling effects were found for a few variables in the following tests: the Cued Recall test (CRT), the Paired Associates Learning (PAL), the Cats and Dogs and the Simple Reaction Time (SRT). These were observed in the CRT for the total immediate recall (A1–A3 total recall) and the total delayed recall, in the PAL for the number of stages completed, in the Cats and Dogs for the correct score, and in the SRT for the percent of correct answers. Regarding floor effects, only a few were detected in the verbal and visual span backwards (Digit and SSP Visual Span) and in the total score of the Weigl Sort Test.

### Cognitive performance in DS participants compared to standard norms

**Figure 1:**
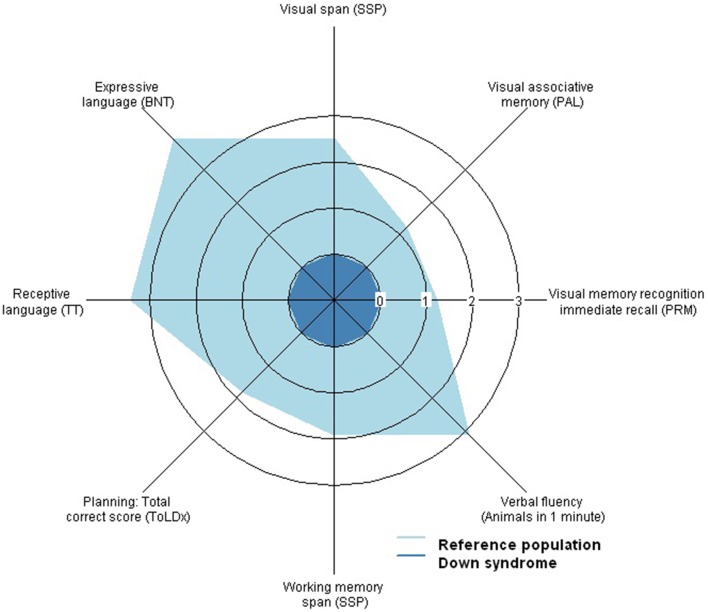
**Radar plot representing the severity of cognitive impairment in Down syndrome (DS) compared to age-matched typically developed adults on attention, memory, language and executive functioning components**. Axis values indicate the absolute value of Cohen's effect size (*d*) for the differences between both populations. For this purpose, the performance of the participants with DS has been standarized to 1 which is equivalent to an effect size of *d* = 0. DS adults show a severe dyfunction of language capacity (|*d*| > 3), a substantial deficit on attention span and executive functions (|*d*| > 1.5) and a moderate deficit in episodic memory(|*d*| > 1).

Descriptive analyses, Cohen effect sizes (*d*), and confidence intervals (95% CI) of cognitive performance in DS and age-matched typically developed adults are summarized in Table 2. Cohen effect sizes on the differences of cognitive performance between DS young adults and euploid subjects revealed the following continuum in the magnitude (*d*) of the deficits in DS: a severe dysfunction of language capacity, a substantial deficit on attention span and executive functions, a moderate deficit in episodic memory and learning abilities, and mild differences in psychomotor speed (Figure 1).

**Table 2:** **Cognitive performance in Down syndrome participants compared to standard norms**.

**Groups**	**Down syndrome**				**Reference standard norms**				**Cohen's-*d*^b^**	**95% CI^c^**
	**Mean (SD)^a^**	**Range (min–max)**	**Age range**	***n***	**Mean (SD)**	**Range (min–max)**	**Age range**	***n***		
**ATTENTION**										
SRT: simple RT latency (ms^d^)	588.0 (220.0)	302–1430	16–34	85	–	–	–	–	–	–
SRT: simple RT(%) correct	96.6 (5.7)	68–100	16–34	85	–	–	–	–	–	–
SSP visual span	3.2 (1.5)	0–6	16–34	86	6.7 (1.3)	3–9	16–39	199	−2.5	−2.8, −2.2
Digit span	2.8 (0.8)	0–4	16–34	86	6.2 (1.0)	4–9	18–34	84	−3.6	−4.1, −3.1
**PSYCHOMOTOR SPEED**										
MOT: mean latency (ms)	1138.0 (391.0)	576–2645	16–34	86	928.0 (254.0)	445–2204	16–39	143	0.7	0.4, 0.9
**VISUAL EPISODIC MEMORY**										
**Visual associative memory**										
PAL: stages completed	6.7 (1.8)	1–8	16–34	85	8.0 (0.04)	7–8	16–39	175	−1.2	−1.5, −0.2
PAL: first trial memory	11.0 (4.8)	0–21	16–34	85	21.6 (3.5)	7–26	16–39	146	−2.6	−3.0, −2.3
PAL: total errors adjusted	70.1 (60.90)	6–213	16–34	85	7. 2 (9.1)	0–82	16–39	168	1.7	1.4, 2.0
**Visual recognition**										
PRM: (%) immediate recall	66.9 (19.3)	25–100	16–34	86	87.8 (12.5)	58.30–100	16–39	51	−1.2	−1.6, −0.8
PRM: (%) delayed recall	61.0 (18.6)	25–100	16–34	85	–	–	–	–	–	–
**EXECUTIVE FUNCTIONS**										
**Verbal fluency**										
Semantic word fluency	9.4 (4.3)	0–21	16–34	85	23.6 (4.9)	9–34	18–34	87	−3.1	−3.5, −2.6
**WORKING MEMORY**										
SSP visual span backwards^e^	2.4 (1.6)	0–8	16–34	85	5.0 (0.9)	3–7	18–34	87	−1.9	−2.3, −1.6
Digit span backwards	1.4 (1.2)	0–3	16–34	86	5.2 (1.3)	3–8	18–34	84	−3.0	−3.4, −2. 6
**Planning^f^**										
ToLDx: total correct Score	1.7 (1.4)	0–5	16–34	82	4.4 (1.7)	–	13–15	76	−1.8	−2.1, −1.4
ToLDx: total move score	84.7 (39.2)	0–170	16–34	82	29.0 (13.5)	–	13–15	76	1.9	1.5, 2.2
ToLDx: probl-solving time (s^g^)	763.0 (289.0)	0–1200	16–34	82	214.7 (98.3)	–	13–15	76	2.5	2.1, 2.9
**LANGUAGE**										
**Comprehension**										
Token Test: total score	19.6 (6.5)	1–35	16–34	85	35.5 (0.7)	33–36	18–34	87	−3.4	−3.9, −3.0
**Naming**										
Boston Naming Test: total score	24.0 (9.5)	0–53	16–34	82	52.4 (4.3)	39–59	18–34	87	−3. 9	−4.4, −3.4

a *Results are presented as mean (standard deviation)*.

b *Cohen's effect size. Differences larger than three pooled standard deviations: |d| > 3); substantial impairment (|d| > 1.5); moderate impairment (|d| > 1); and mild impairment (|d| > 0.5)*.

c *Confidence Interval*.

d *Milliseconds*.

e *Results are compared to standard norms from the Corsi Block provided by the NEURONORMA young adults Project*.

f *Results are compared to standard norms from adolescent typically developed subjects, ages 13–15 years*.

g *Seconds*.

### Impact of intellectual quotient (IQ), gender, and age on cognitive performance and functional outcomes

ANCOVA models were applied to analyze effects of IQ, gender, and age on the baseline cognitive performance of DS participants, adjusting for co-variables (Tables 3–5). IQ was related to the significant (*p* < 0.05) differences in measures of cognitive capacity between participants of IQ < 40 and those of IQ ≥ 40, with the exception of performance on the SRT, Digit Span Backwards and the Weigl Sort Test. These assessing reaction time, verbal working memory and mental flexibility, respectively. As expected, in all cases higher IQ levels were associated with greater cognitive attainment irrespective of chronological age or gender (i.e., comparing subjects of equal age and gender). In addition, significant effects of IQ level were observed in adaptive behavior in most functional skill areas assessed with the ABAS-II such as Communication, Community Use, Functional Academics, Home Living, Health and Safety, Self-Direction, Social Skills, and ABAS total score (*p* < 0.05). Once again, those participants with higher IQ showed better outcomes in adaptive behavior and thus better competence in daily living. However, no significant effect of IQ emerged on the Kidscreen-27 (*p* > 0.05) which assessed different aspects of quality of life.

**Table 3:** **Impact of intellectual quotient, gender, and age on attention, psychomotor, memory, and language performance in Down syndrome participants**.

**Down syndrome**	**Intelligence quotient (<40 vs. ≥ 40)**			**Gender (female vs. male)**			**Age**		
	**Estimate^a^**	**95% CI^b^**	***p*-values**	**Estimate^c^**	**95% CI**	***p*-values**	**Estimate^d^**	**95% CI**	***p*-values**
**ATTENTION**									
SRT: simple RT latency (ms^e^)	57.2	[−39.1; 153.6]	0.24	−37.7	[−134.2; 58.8]	0.44	8.68	[39.1; 153.6]	0.13
SRT: simple RT(%) correct	−1.9	[−4.3; 0.5]	0.12	2.8	[0.3; 5.2]	0.02^*^	−0.25	[−0.5; 0.03]	0.08
SSP visual span	−0.8	[−1.4;- 0.2]	0.01^*^	0.7	[0.1; 1.3]	0.02^*^	−0.03	[−0.0; 0.03]	0.34
Digit span	−0.4	[−0.7;- 0.03]	0.03^*^	0.3	[0.01; 0.7]	0.04^*^	0.02	[−0.01; 0.06]	0.20
**PSYCHOMOTOR SPEED**									
MOT: mean latency (ms)	176.1	[9.3; 342.9]	0.03^*^	3.6	[−164.2; 171.4]	0.96	15.7	[−4.2; 35.5]	0.12
**EPISODIC MEMORY**									
**Visual associative memory**									
PAL: stages completed	−1.0	[−1.8;-0.3]	0.006^**^	1.30	[0.6; 2.0]	0.001^**^	0.02	[−0.06; 0.10]	0.61
PAL: first trial memory	−1.8	[−3.7; 0.2]	0.07^+^	3.47	[1.5; 5.4]	0.001^**^	0.002	[−0.2; 0.2]	0.98
PAL: total errors adjusted	35.1	[11.8; 58.4]	0.004^**^	−52.24	[−75.6;-28.9]	<0.001^***^	0.1	[−2.7; 2.9]	0.94
**Visual recognition**									
PRM: (%) immediate recall	−12.1	[−20.2; −4.1]	0.004^**^	4.25	[−3.8; 12.3]	0.29	−0.6	[−1.6; 0.3]	0.20
PRM: (%) delayed recall	−8.0	[−16.0; 0.01]	0.05^+^	2.04	[−6.0; 10.1]	0.61	−0.7	[−1.6; 0.3]	0.15
**Verbal episodic memory**									
CRT:A1-A3 free immediate recall	−2.5	[−5.0; 0.1]	0.05^+^	3.6	[1.0; 6.1]	0.007^**^	0.1	[−0.2; 0.4]	0.68
CRT:A1-A3 total immediate recall	−0.7	[−1.5; 0.2]	0.11	0.8	[−0.01; 1.7]	0.05^+^	0.03	[−0.1; 0.1]	0.48
CRT: free delayed recall	−0.9	[−2.0; 0.2]	0.11	1.5	[0.4; 2.6]	0.008^**^	0.1	[−0.1; 0.2]	0.33
CRT: total delayed recall	−0.1	[0.4; 0.2]	0.45	0.05	[−0.2; 0.3]	0.68	0.02	[−0.01; 0.05]	0.24
**LANGUAGE**									
**Comprehension**									
Token Test: total score	−5.6	[−8.1; −3.0]	<0.001^***^	3.25	[0.7; 5.8]	0.01^*^	0.01	[−0.3; 0.3]	0.97
**Naming**									
Boston Naming Test: total score	−9.9	[−13.6; −6.1]	<0.001^***^	2.22	[−1.5; 5.9]	0.2	−0.1	[−0.6; 0.3]	0.59

a *Estimated mean difference between persons with DS with IQ < 40 and persons with DS with IQ ≥ 40 adjusted for gender and age*.

b *Confidence Interval*.

c *Estimated mean differences between female and male persons with DS adjusted for IQ and age*.

d *Estimated mean differences associated to one year of age difference in persons with DS adjusted for IQ and gender*.

e *Milliseconds*.

* *Significant estimated effects of the variable of interest (p<0.05)*.

** *Significant estimated effects of the variable of interest (p<0.01)*.

*** *Significant estimated effects of the variable of interest (p<0.001)*.

+ *Marginal non-significant estimated effects of the variable of interest*.

**Table 4:** **Impact of intellectual quotient (IQ), gender, and age on executive functioning in Down syndrome participants**.

	**Down syndrome**								
	**Intelligence Quotient (<40 vs. ≥ 40)**			**Gender (female vs. male)**			**Age**		
	**Estimate^a^**	**95% CI^b^**	***p*-values**	**Estimate^c^**	**95% CI**	***p*-values**	**Estimate^d^**	**95% CI**	***p*-values**
**EXECUTIVE FUNCTIONS**									
**Verbal fluency**								
Semantic word fluency	−2.6	[−4.4; −0.8]	0.006^**^	−0.1	[−1.9; 1.7]	0.87	0.1	[−0.1; 0.3]	0.29
**Working memory**								
SSP visual span backwards	−0.8	[−1.4; −0.1]	0.02^*^	0.7	[0.05; 1.4]	0.03^*^	0.03	[−0.04; 0.1]	0.42
Digit span backwards	−0.3	[0.86; 0.15]	0.16	0.43	[−0.1; 0.9]	0.09	−0.01	[0.04; 0.6]	0.64
**Planning**								
ToLDx: total correct score	−1.0	[−1.6; −0.5]	<0.001^***^	0.9	[0.4; 1.5]	0.002^**^	−0.03	[−0.1; 0.03]	0.36
ToLDx: total move score	26.3	[10.0; 42.7]	0.002^**^	−20.9	[−37.2; −4.6]	0.01^*^	0.9	[−1.0; 2.8]	0.33
ToLDx: problem-solving time (s^e^)	180.3	[58.2; 302.4]	0.004^**^	−143.9	[−265.6; −22.1]	0.02^*^	9.2	[−5. 1; 23.5]	0.20
**Mental flexibility**								
Weigl sort test: total score	−0.3	[−1.0; 0.3]	0.32	1.2	[0.5; 1.9]	0.001^**^	−0.02	[−0.1; 0.1]	0.62
**Inhibition**								
Cats and dogs: total time (s)	7.6	[0.6; 14.7]	0.03^*^	−2.4	[−9. 5; 4.6]	0.48	0.2	[−0.7; 1.0]	0.66
Cats and dogs: correct score	−0.6	[−1.1; −0.1]	0.02^*^	−0.01	[−0.5; 0.5]	0.96	−0.02	[0.1; 0.03]	0.44

a *Estimated mean difference between persons with DS with IQ < 40 and persons with DS with IQ ≥ 40 adjusted for gender and age*.

b *Confidence Interval*.

c *Estimated mean differences between female and male persons with DS adjusted for IQ and age*.

d *Estimated mean differences associated to one year of age difference in persons with DS adjusted for IQ and gender*.

e *Seconds*.

* *Significant estimated effects of the variable of interest (p < 0.05)*.

** *Significant estimated effects of the variable of interest (p < 0.01)*.

*** *Significant estimated effects of the variable of interest (p < 0.001)*.

**Table 5:** **Impact of intellectual quotient (IQ), gender, and age on functional outcomes in Down syndrome participants**.

	**Down syndrome**								
	**Intelligence Quotient (<40 vs. ≥ 40)**			**Gender (female vs. male)**			**Age**		
	**Estimate^a^**	**95% CI^b^**	***p*-values**	**Estimate^c^**	**95% CI**	***p*-values**	**Estimate^d^**	**95% CI**	***p*-values**
**ADAPTIVE BEHAVIOR**									
ABAS-Communication	−8.7	[−13.9; −3.6]	0.001^**^	4.2	[−1.0; 9.4]	0.11	0.1	[−0.7; 0.8]	0.82
ABAS-community use	−9.7	[−15.0; −4.4]	<0.001^***^	2.8	[−2.5; 8.1]	0.29	0.5	[−1.1; 1.1]	0.13
ABAS-functional academics	−13.3	[−20.4; −6.3]	<0.001^***^	9.0	[1.9; 16.1]	0.01^*^	0.1	[−0.1; 1.0]	0.77
ABAS-home living	−5.6	[−10.6; −0.6]	0.02^*^	4.2	[−0.8; 9.3]	0.09	0.4	[−0.2; 1.0]	0.17
ABAS-health and safety	−5.8	[−9.7; −1.8]	0.005^**^	0.5	[−3.5; 4.5]	0.81	0.2	[−0.3; 0.7]	0.42
ABAS-leisure	−2.5	[−7.6; 2.5]	0.32	1.2	[−3.8; 6.3]	0.62	−0.2	[−0.8; 0.4]	0.50
ABAS-self-care	−1.5	[−5.1; 2.2]	0.42	2.4	[−1.3; 6.0]	0.20	0.03	[−0.4; 0.5]	0.85
ABAS-self-direction	−8.7	[−14.8; −2.5]	0.006^**^	4. 9	[−1.2; 11.0]	0.11	0.2	[−0.6; 0.9]	0.64
ABAS-social skills	−7.6	[−12.4; −2.8]	0.002^**^	1.8	[−2.9; 6.6]	0.44	−0.5	[−1.0; 0.1]	0.09
ABAS-work	–		–	–		–	–		–
ABAS-total score	−63.4	[−100.4; −26.4]	0.001^**^	31.1	[−6.1; 68.3]	0.10	0.8	[−3.6; 5.2]	0.71
**QUALITY OF LIFE**									
Kidscreen 27-physical	0.6	[−1.1; −2.2]	0.49	−1.7	[−3.3; −0.05]	0.04^*^	−0.2	[−0.4; 0.03]	0.09
Kidscreen 27-psychological	0.5	[−1.2; −2.3]	0.53	0.2	[−1.5; 1.9]	0.82	−0.2	[−0.4; −0.01]	0.03^*^
Kidscreen 27-autonomy and parents	0.1	[−1.1; 1.4]	0.80	1	[−0.3; 2.3]	0.11	−0.05	[−0.2; 0.1]	0.46
Kidscreen 27-peers and social	0.5	[−1.4; 2.4]	0.60	0.9	[−1.0; 2.8]	0.35	−0.05	[−0.3; 0.2]	0.66
Kidscreen 27-school	–	–	–	–	–	–
Kidscreen 27-total score	2.8	[−3.9; 9.6]	0.39	−1.0	[−7.6; 5.6]	0.75	−0.9	[−1.7; 0.1]	0.03^*^

a *Estimated mean difference between persons with DS with IQ < 40 and persons with DS with IQ ≥ 40 adjusted for gender and age*.

b *Confidence Interval*.

c *Estimated mean differences between female and male persons with DS adjusted for IQ and age*.

d *Estimated mean differences associated to one year of age difference in persons with DS adjusted for IQ and gender*.

* *Significant estimated effects of the variable of interest (p < 0.05)*.

** *Significant estimated effects of the variable of interest (p < 0.01)*.

*** *Significant estimated effects of the variable of interest (p < 0.001)*.

**Figure 2:**
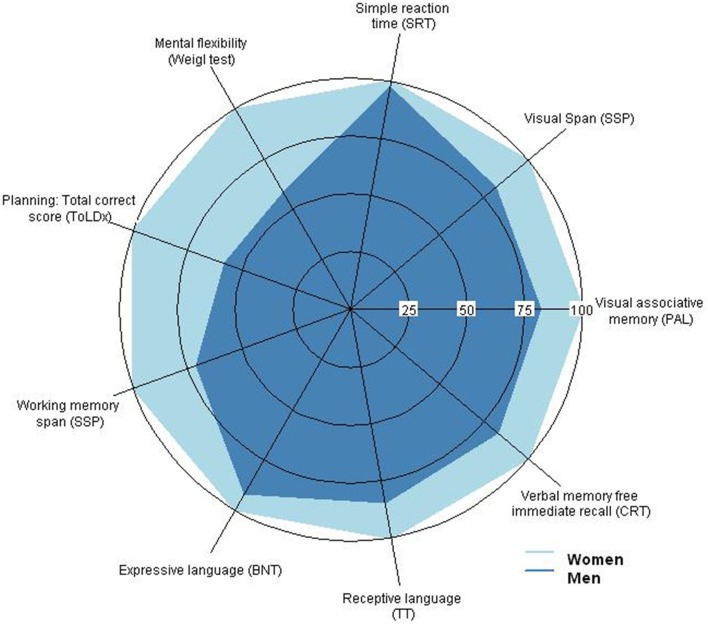
**Radar plot representing the statistically significant differences in cognitive performance between men and women with Down Syndrome (DS) on attention, memory, language and executive functioning components**. Axis values indicate the performance in percentage relative to the women's performance, which has been set to 100%. Men with DS performed significantly poorer than women in all four cognitive domains.

Concerning gender, significant differences between men and women were mainly observed in cognitive performance and less in functional outcomes. Women performed significantly better than men of the same age and IQ in most cognitive tests (Tables 3–5), with the most consistent differences occurring in episodic memory and executive functioning (Figure 2). Women also responded better in episodic memory tests, in particular visual associative memory (PAL) and free recall of verbal information (CRT) (*p* < 0.05), but not in visual memory recognition (PRM; *p* > 0.05). Concerning executive functions, women showed significantly better performance (*p* < 0.05) in cognitive flexibility and planning. Furthermore, they exhibited higher scores in receptive language and attention measures of span capacity, and better accuracy in the simple reaction time task (*p* < 0.05). Gender-related differences were also observed in the functional domain, with women having a significantly better performance than men in adaptive behavior, specifically in Functional Academic (emergent literacy and numeracy basics for current life use) (*p* < 0.05), but described lower health perception regarding their physical wellbeing as reported by parents on the Kidscreen-27 (Kidscreen 27-Physical; *p* = 0.04). Overall, these results indicate that gender exerts significant effects on cognitive and functional capacities in DS participants, favoring women against men in cognitive functioning and adaptive skills but not in QoL.

Significant negative trends rarely emerged on quality of life outcomes linked to the effect of age in DS participants after adjusting for IQ and gender. Age did not affect adaptive behaviors, nor most measures of quality of life significantly. However, age did affect psychological well-being, which affected total quality of life (*p* < 0.03). Parents responding to the Kidscreen 27 Psychological and Total score items indicated poorer psychological wellbeing and overall health perception as the children grew older.

### Relationship between cognitive deficits and functional outcome

The Pearson correlation coefficient was calculated to assess the relationships between cognition and functionality, in order to identify meaningful cognitive measures of potential change for clinical trials. Moderate associations emerged among a wide spectrum of cognitive measures and IQ with specific adaptive skills, or the total score in the ABAS-II, while no association was detected with quality of life measures.

The strongest associations were found between cognitive performance and functional academic skills (ABAS-II). Positive associations emerged between Functional Academics and measures of receptive and expressive language (Token Test: *r* = 0.65, [0.51, 0.76]; Boston Naming: *r* = 0.42, [0.22, 0.58]) and executive components of verbal fluency (Semantic word fluency: *r* = 0.40, [0.20, 0.56]). Positive associations were also found for working memory for visual and verbal information (SSP span backwards: *r* = 0.47, [0.29, 0.62]; Digit span backwards: *r* = 0.48, [0.30, 0.63]), planning (ToLDx Total correct score: *r* = 0.53, [0.35, 0.67]), attention span for visual and verbal information (SSP span: *r* = 0.56, [95%-CI: 0.39, 0.69]; Digit span: *r* = 0.46, [0.28, 0.62]), and memory recognition for immediate and delayed recall of visual information (PRM (%) immediate recall: *r* = 0.45, [0.26, 0.60]; PRM (%) delayed recall: *r* = 0.48, [0.29, 0.63]). Negative associations were found between Functional Academics and error rate in the visual associative learning task (PAL total errors adjusted: *r* = −0.56, [−0.69, −0.39]) and planning accuracy deficits (ToLDx Total move score: *r* = −0.51, [−0.66, −0.33]). These results indicate that higher attainment in functional academic skills (emergent literacy and numeracy basics for current life use) could be strongly linked to a more efficient overall cognitive functioning in DS participants. In addition, a positive consistent association emerged between Functional Academics and IQ (IQ: *r* = 0.52, [0.35, 0.66]). These results confirm previous assumptions, and suggest that specific cognitive measures are potentially good end-point measures for estimating changes in functional outcome in clinical trials.

Communication and Community use subscales of the ABAS-II also correlated consistently with cognitive attainment. Positive correlations were found between communicative abilities and visual attention span (SSP span: *r* = 0.40, [0.20, 0.56]), receptive and expressive language (Token Test: *r* = 0.52, [0.34, 0.66]; Boston Naming Test: *r* = 0.41, [0.21, 0.58]). In addition, a negative association was observed between ability to communicate and the number of errors performed during visual associative learning (PAL total errors adjusted: *r* = −0.46, [−0.61, −0.27]). Community use was mainly related to cognitive measures of receptive language (Token Test: *r* = 0.52, [0.30, 0.63]) and executive components of working memory for visual and verbal information (SSP span backwards: *r* = 0.43, [0.24, 0.59]; Digit span backwards: *r* = 0.41, [0.21, 0.57]) and planning (ToLDx Total correct score: *r* = 0.40, [0.19, 0.56]). In all cases, a higher performance in specific cognitive tests was consistently related to a greater ability to communicate in daily life and higher independent functioning within the community.

Finally, language comprehension emerged as having the most consistent association with the overall score in adaptive behavior (ABAS Total Score) (Token Test: *r* = 0.52, [0.35, 0.66]). Other cognitive measures were consistently correlated with the ABAS Total Score such as visual attention span (SSP span: *r* = 0.40, [0.20, 0.56]) and executive components of visual working memory (SSP span backwards: *r* = 0.41, [0.22, 0.57]) and planning (ToLDx Total correct score: *r* = 0.41, [0.21, 0.58]; ToLDx Total move score: *r* = −0.48, [−0.63, −0.29]). These results indicate that better language comprehension, attention, and executive functioning are the cognitive capacities more closely related to higher competence in overall adaptive skills and, therefore, in everyday life independence for DS participants.

## Discussion

This study proposes a new neurocognitive battery for clinical trials in DS adults (the TESDAD battery), using chronologically age-matched fully-developed subjects for comparison as a more useful approach for the characterization of the DS cognitive profile. This battery also provides clinically useful measures closely linked to prefrontal-temporal brain networks and to functional competence in everyday life following interventional studies. Finally, our study emphasizes the need to determine the modulation effects of intellectual quotient, gender, and age on cognitive treatments.

### Magnitude of cognitive deficits in DS adults

The results of this study support the demonstration (Abbeduto et al., 2001; Laws and Bishop, 2004; Næss et al., 2011) that language impairment is the strongest cognitive disturbance in young DS adults with receptive abilities being more preserved than expressive skills. In addition, and as previously reported, the relative strength of visuospatial processing over verbal tasks suggests that language impairment is the primary landmark of global intellectual impairment in DS (Lanfranchi et al., 2004; Edgin et al., 2010b). After language, attention and executive functions differed more from standard norms, with verbal span capacity and verbal fluency presenting the strongest deficiencies, followed by working memory; in contrast, planning was relatively more preserved. These results concur with the portrayal of a broad, marked dysexecutive syndrome in DS (Rowe et al., 2006; Lanfranchi et al., 2010) probably due to the reduced size of the prefrontal cortex (Contestabile et al., 2010; Lott and Dierssen, 2010), in particular of the anterior cingulate gyrus, medial, and dorsolateral prefrontal cortices as reported in neuroimaging studies of DS adults (Raz et al., 1995; White et al., 2003; Carducci et al., 2013). Areas such as these actively contribute to mnemonic processing and executive control in euploid subjects (Braver, 2001; Wager and Smith, 2003; Blumenfeld et al., 2011), thus generalized impairment of high order frontal-dependent processes, together with language, represent a crucial target for therapeutic intervention in DS.

Overall performance in episodic memory was also poor although superior to language, attention, and executive functions. It is noteworthy that our results showed a better preservation of hippocampal-dependent memory processes, such as storage and consolidation, compared to frontal-mediated processes (information coding, retrieval strategies and attention control) in DS. Findings substantiated by the higher performance exhibited in the recognition and cued recall trials as compared to free recall, and by the higher ratio of perseverative errors compared to intrusions in the verbal learning task (see Supplementary Results). This mnemonic profile indicates that poor monitoring and executive control, rather than storage difficulties, are mainly responsible for poor memory performance. In this regard, structural neuroimaging studies have related impaired memory performance in DS adults with reductions in the prefrontal, hippocampus, and parahippocampal areas of these subjects (Krasuski et al., 2002; Teipel et al., 2004; Beacher et al., 2005). Postmortem histological studies have, moreover, consistently demonstrated that the dendritic morphology of hippocampal neurons is compromised in DS adult brains (Ferrer and Gullotta, 1990; Takashima et al., 1994). In summary, our results indicate executive dysfunction as a major factor underlying memory impairment in DS. Thus, effective therapies targeting prefrontal-dependent executive functions in this population would enhance cognitive performance.

### Effects of IQ, gender, and age on cognitive and functional outcomes in DS

We explored the association of clinical and sociodemographic variables such as IQ, gender, and age with cognitive and functional performance in DS. Our regression analyses, in concurrence with other authors, revealed that the explanation for the extensive variability found in the neurocognitive performance of DS adults lies in the primary variable of the IQ level. The most consistent associations with IQ were found with language, its use in everyday functioning (learning of literacy basics, communication skills, social abilities, and efficient use of community resources), and with global adaptive competence. No effect of IQ was observed, however, on quality of life outcomes. A finding that could partly be explained by the fact that in euploids, emotional aspects are more closely related to QoL perception than IQ (Takeuchi et al., 2014). The use of parent-proxy measures for determining QoL perception in DS is, nevertheless, a surrogate and a probably biased outcome based on QoL self-perception in these subjects.

It is noteworthy that gender showed a widespread influence on cognitive variables whilst its impact on functional outcomes was minor. From our analyses we can conclude that men with DS perform at a significantly poorer level than DS women, in particular with respect to episodic memory and executive processing. They also exhibit poorer functional academic skills in everyday life, but present a higher QoL perception concerning their physical well-being. Although the differences observed in cognitive performance between genders are mild, they may explain the higher IQ level and better competence exhibited by women in everyday functioning, in particular related to command of language. Other studies have also reported that women with trisomy 21 display a higher level of cognitive and adaptive functioning than DS men (Lund, 1988; Määttä et al., 2006). Taken together, these findings suggest that gender may exert a relevant modulating effect on cognitive functioning in DS participants favoring women, which is not the case in healthy participants. The poorer QoL status in young women with DS compared to men, especially with respect to their physical well-being, may not be characteristic of DS associated with gender, since it has also been reported in woman from euploid population (Torsheim et al., 2006; Michel et al., 2009).

The impact of age on neurocognitive outcomes was negligible and restricted to QoL perception. In a similar manner to healthy adolescents and young adults, increasing age in DS participants was associated with a decline in QoL, in spite of the fact that women reported poorer outcomes compared to men (Bisegger et al., 2005; Michel et al., 2009). Thus, lower QoL with increasing age is not a distinctive trend in DS. No significant impact of age was found on cognitive and adaptive behavior outcomes, probably due to the age range of our sample (16–34 years old), representative of late adolescence and adulthood when the negative consequences of premature aging upon cognition and everyday life competence have not yet been detected. Our results suggest that during this period overall cognitive capacity in DS adults has probably reached a plateau, similar to the scenario of normally developed adults who reach their peak performance between 18 and 30 years of age (Peña-Casanova et al., 2012). Taken together, our results emphasize the need to explore the modulating effects of IQ, gender, and age on cognitive enhancing treatments in the DS population.

### Relationship to functional outcome in DS participants for interventional studies

We explored the associations between cognitive performance, IQ, and functional outcomes of adaptive behavior and QoL in DS. The aim was to identify specific relationships between cognitive performance and different aspects of functional outcome that could potentially serve for expecting functional change following interventional studies. Cognitive-related outcomes were closely linked to functional aspects of language and global adaptive competence in everyday life. It is worth mentioning that auditory comprehension and functional academic measures have a great potential as end-point measures of therapeutic intervention for clinical trials: the former as a cognitive key target for therapeutic intervention, and the latter as a primary functional outcome measure of clinical efficacy.

According to the results obtained in the regression analysis, it could be argued that IQ could be a good predictor of functional outcome for longitudinal interventional studies. Specific cognitive capacities, however, showed consistent associations with functional outcomes in the univariate analysis. IQ remains stable during adult life whilst cognitive capacities underlying intellectual status, such as attention, memory, language, and executive functions, are dynamic throughout the lifespan. These changes in cognitive capacity provide greater sensitivity for assessing the efficacy of therapeutic interventions. In addition, these cognitive capacities can be precisely measured with specific tests that are sensitive to clinical and subclinical changes. The fact that these cognitive measures are considered a proxy of such subclinical changes, closely related with the abnormal functioning of prefrontal-temporal brain networks, is extremely important when testing new therapeutic strategies for mental disability. Currently, a major caveat of clinical trials targeting functional change in DS is that follow-up periods tend to be too short (less than 12 months on average), while improvements in complex functional skills in DS require longer periods (Costa, 2011; Boada et al., 2012) We agree with this view but suggest that subclinical cognitive gains related to positive pharmacological and/or behavioral interventions in DS may be sufficient for a mild, but significant, impact on everyday life functioning, similar to what we can expect in other pathological conditions such as AD (Insel et al., 2013). Studies with extended follow-up periods under active treatment are needed to probe our hypotheses and ensure the validity of the proposed linkages as clinically meaningful for estimating functional change in interventional studies.

### Limitations

Several limitations should be considered when interpreting the results of our study. First, the neurocognitive assessment tools we employed may have influenced the DS cognitive profile observed. The majority of the tests included in our TESDAD battery are, nevertheless, recognized as valid and feasible for tracking cognitive deficits in pathological conditions (Ersche et al., 2012; Juncos-Rabadán et al., 2014), they are well standardized and extensively normalized, and acceptable for DS participants with mild to moderate mental disability (Devenny et al., 2002; Ball et al., 2008; De la Torre et al., 2014). Floor effects were observed for the verbal and visual backward span and mental flexibility tasks, whereas ceiling effects where shown in a few episodic memory variables and in the response inhibition test regarding task accuracy but not for time of response. These limitations were found mainly in specific tests developed for mentally disabled individuals (Weigl, Cats and Dogs and CRT). Although, these findings suggest that these tasks could be replaced, we can still consider them suitable for the assessment of the selected population due that these were not found for all variables in each of the mentioned tests (e.g., free recall measures in the CRT), or provide an affordable evaluation of high-order executive capacities in intellectual disability (e.g., Weigl). One of the few commonly used cognitive batteries is the Arizona Battery (ACTB), developed for school age children and young adults with DS (age range 7–30 years), employing the mental-age matched procedure (Edgin et al., 2010a). The principal differences between the ACTB and the TESDAD battery are that TESDAD allows a more thorough, direct cognitive assessment of the main mnemonic and executive components with language being a key domain, whereas ACTB includes a deeper assessment of executive-behavioral dysfunction using questionnaires for parents. In addition, the TESDAD was also designed to be sensitive to mild cognitive impairment, making this tool potentially valid for capturing deterioration in the prodromal stage. Another limitation is that the TESDAD only explores “cool-cognitive functions,” whereas “hot-cognitive processing” involving emotional, motivational, and rewarding aspects are omitted. We focused our assessment on cool-conscious high reasoning processes, in particular on executive and mnemonic processing supported by the hippocampus and frontal cortices, because preclinical and clinical evidence consider those to be critical targets for therapeutic intervention in DS. Nonetheless, the TESDAD Battery should undergo further modifications to integrate new feedback provided by future preclinical and clinical evidence. Another drawback is that IQ estimation within the lowest range (IQ < 40) could not be exactly determined with the K-BIT. In addition, the fact that the Kidscreen-27 questionnaire was designed for assessing younger individuals (8–18 years of age), may have partly compromised its sensitivity for determining QoL perception in young adults. The lack of an overall composite score integrating cognitive and functional outcomes is another important limitation, ongoing issue for the TESDAD battery. This comprehensive score would be a valuable asset for globally evaluating treatment effects in longitudinal studies. Finally, the lack of a test-retest reliability assessment of the overall battery is another important drawback. Nonetheless, the selection of tests was based upon previous reliability studies carried for each of these tools (Strauss et al., 2006).

## Conclusion

In summary, the TESDAD battery is a useful tool for a standardized neurocognitive assessment of DS in clinical trials. The most relevant features of this battery include a chronological age-matched approach, high sensitivity for detecting mild to moderate cognitive deficits, and a strong relationship to clinically relevant functional measures. These features make the battery suitable for capturing changes derived from therapy which allow its efficacy to be established.

### Conflict of interest statement

The authors declare that the research was conducted in the absence of any commercial or financial relationships that could be construed as a potential conflict of interest.

## Footnotes

^1^Tables for the Cantab test battery are provided in electronic format alongside the product as standard. This allows the replicability of the present data reported in this paper. Due to the fact that norms are copyrighted material, these can't be reproduced in the present study. In the specific case of the Cantab tests, the papers cited report only part of the normative sample included in the current norms, therefore do not correspond precisely to the figures. The present figures correspond to the current Cantab norms, as available with the test.
