# Supplementary material for: A new cognitive evaluation battery for Down syndrome and its relevance for clinical trials
Source: Front Psychol. 2015 Jun 4;6:708. doi: 10.3389/fpsyg.2015.00708 (PMC4455308; doi:10.3389/fpsyg.2015.00708)
Supplement: Supplementary file 1 [file DataSheet1.PDF]

Supplemental Materials

A.1. The TESDAD Battery

A.1.1 Cognitive assessment and intellectual quotient estimation (IQ)

Intellectual quotient

The Kaufman Brief Intelligence Test (K-BIT, Spanish version, Kaufman, 1990, 1997), This test evaluates the intellectual status of individuals covering a wide age range, from 4 to 90 years, and significantly correlates with the WISC-III (Canivez et al., 2005). The intellectual quotient corresponds to the K-BIT standardized total score and was considered in the analyses to control for clinically relevant pre-existing differences in general intellectual disability among individuals.

Psychomotor speed

Motor Screening test (MOT, CANTAB® Battery; (Buffalo et al., 2006)(Robbins and Sahakian, 1996). Participants are instructed to touch a series of crosses that appear randomly on the screen. This task assesses psychomotor speed and accuracy. The measure of response latency (in milliseconds) was considered in the present study.

Attention

Simple Reaction Time (SRT; CANTAB® Battery). In this test, subjects must press the button on a press pad as soon as they see a square appearing in the middle of the screen. Intervals between the examinee's response and the onset of the next stimulus are variable during task performance. This task tests general alertness and motor speed. Measures of accuracy (number of correct trials; range: 0-100) and response latency (in milliseconds) were used for the analyses.
Digit Span: forward recall (WAIS-III subtest (Wechsler, 1997), Spanish version. Subjects are required to listen to a series of numbers with randomized presentation, and then repeat them back. The length of the series increases with the subject's success. Forward recall score provides a good measure of verbal attention and memory span.
Spatial Span: forward recall (SSP; CANTAB® Battery). Subjects must memorize and reproduce a sequence of spatial locations demonstrated on the computer screen. Participants view a sequence of squares that change, one at a time, from white to a different color. The examinee has to touch on the screen the squares in the same order as they were presented. Length of the sequence increases with the subject's success. Forward recall is predominantly a measure of visual attention and memory span. Measures of span length and total number of errors were considered for the analyses.

Executive functions

Digit Span: backward recall (WAIS-III subtest (Wechsler, 1997), Spanish version 1999). Subjects are required to listen to a series of numbers with randomized presentation, and then repeat them backwards. The length of the series increases with

the subject's success. Backward recall score is predominantly a measure of verbal working memory span.
Spatial Span: backward recall (SSP; CANTAB® Battery). Subjects are required to memorize and inversely reproduce a sequence of spatial locations that appear on the computer screen. The examinee has to reproduce this sequence backwards by touching items in the inverse order they were originally presented. Length of the sequence increases with the subject's success. Backward recall is predominantly a measure of visual working memory span. Measures of span length and total number of errors were used for the analyses.

Word fluency (Benton et al., 1976). Subjects are asked to generate as many words as possible in 1 minute belonging to the specified category of "animals". As in the previous test, high scores indicate greater verbal fluency ability.

Tower of London-Drexel University (ToLDx; Culbertson & Zillmer, 2005). For this study we used the pediatric version (ages 7 to 15) in order to avoid floor effects. Standard norms were used from this version from the upper age range: 13 to 15 years old. This test requires the movement of three different colored balls across three different sized pegs in order to replicate a goal configuration. Movements follow strict rules. The first two problems were training tasks, following which 10 problems of increasing complexity were presented. The task ceased after the examinee failed to solve two consecutive problems. For those subjects who failed to complete the entire test, number of movements and problem-solving time were adjusted for the number of uncompleted trials to allow a feasible comparison with higher performers, assigning the maximum number of movements (20) and maximum problem-solving time (120 s) to each failed item. Scores for total problem-solving time (in seconds), total number of moves needed to complete the configuration, and total number of problems solved within the minimum number of moves were used for the analyses, providing a good measure of planning ability.

Weigl Color-Form Sort Test (Goldstein and Scheerer, 1953). This is a set-shifting task that assesses the ability to categorize across two dimensions: color and shape. Instructions for administration and scoring were taken from Strauss & Lewin, 1982(Strauss and Lewin, 1982). Test material consists of 12 tokens: four circles, four triangles, and four squares, shapes are colored blue, red, yellow or green. The 12 tokens are displayed unsorted in front of the examinee. In the first trial the examinee is required to sort the tokens in a way that they go together (color or shape). After this first trial, the examinee is required to sort them again but using a different combination. The examinee receives prompting from the examiner if he or she is not able to change the sorting principle used in the first trail. Scores range from 0 to 5. A maximum score of 5 points is obtained when the subject is able to shift the initial category without a prompt, whereas 0 points are obtained if the subject fails despite prompting. A higher score indicates greater capacity for set-shifting and is considered a good estimation of cognitive flexibility or reversal learning in individuals with intellectual disability.

Cats & Dogs Test (Ball et al., 2008). In this test, a sequence of 16 pictures, 8 cats and 8 dogs arranged in a prefixed order, are presented to the examinee on a single strip of card. The task consists of two trials with two different conditions: a control and an experimental-inhibition trial. In the control the examinee is required to point to each picture in turn and name the animal as quickly as possible. In the experimental-

inhibition condition, the examinee is instructed to say 'dog' when he or she points to a cat and to say 'cat' when pointing to a dog. A practice trial is given to the examinee before the performance of both trial conditions. Measures of task accuracy in the experimental inhibition trial (total number of correct responses) and total time performance (in seconds) were included in the analyses.

Memory and learning

Paired Associates Learning (PAL; CANTAB® Battery). Participants are required to learn associations between an abstract visual pattern and its location. In this task the participant is presented with a number of white boxes, arranged in a circle around an empty central space in the screen. When the trial begins, each of the boxes "opens" in turn to reveal what is underneath. In some cases, the box is empty, while in others a unique abstract pattern is presented. Each of the boxes opens in a randomized order until the participant has looked under every one. Next, a single pattern is presented in the center of the screen and the subject is instructed to touch the box where that pattern has been shown during the presentation phase of the trial. This task increases in difficulty from 1 to 8 patterns. The number of stages correctly completed provides an index of visual episodic memory. Other measures included in the analyses were the total number of errors committed after being adjusted for the number of trials completed, and first trial memory score which measures the number of patterns correctly located after the first trial across all stages completed (ranging from 0 to 26).

Pattern Recognition Memory (PRM, CANTAB® Battery). Participants are shown a series of two blocks of 12 abstract visual patterns, presented sequentially in the center of the computer screen. Patterns are designed so that they cannot easily be given verbal labels. Each pattern is shown for 3 seconds. In each of the 12 recognition trials, two patterns are presented: one from the series that the participants have already seen and another novel pattern. The participant should recognize the pattern that he or she has already previously seen. This same procedure is repeated with a second block of 12 new patterns. The recognition trials, however, commence 20 minutes after the presentation of this second block in order to provide a measure of delayed recall. Measure of correct responses expressed as a percentage (total percent correct) was recorded in this recognition task for immediate and delayed recall.

Cued Recall Test (CRT; Devenny, Zimmerli, Kittler, & Krinsky-McHale, 2002). The test consists of a list of 12 items which should be verbally recalled by the examinee during 3 trials of free and cued recall. The test starts with a learning phase where the examinee is required to learn the list of 12 items using 12 images. Four pictures are presented at a time, one in each quadrant of a card. First, the examinee has to name each of the four pictures in the card, and secondly assign each picture according to a verbal category-cue given by the examiner. In order to assure that the items are correctly encoded, the card is removed and the examinee is asked to immediately recall the four test items by memory. Presentation of the cards during the learning phase cease when the examinee is able to recall all four items correctly from the three cards. The testing phase immediately follows and consists of the three trials of free and cued recall. Before every free recall trial, a short interference task is performed which consists of counting up to 20 for 20 seconds. After this interference task, the examinee is requested to verbally recall as many items from the list, in any order, as he or she is able to remember. Next, the missed items are requested again with a verbal cue for each item

provided. If the examinee fails to retrieve the item despite prompting, he or she is reminded of the missed item in order to allow learning for the next trial. The same procedure is applied for each of the 3 trials. After 20 minutes, a free recall of the list of words is requested, again followed by a cued recall for those words that were not recalled. Measures of immediate free total recall (in the three trials), total immediate recall (free and cued), free delayed recall (after 20 minutes), and total delayed recall (free and cued) were considered in the study as good estimations of verbal episodic memory and for tracking its potential impairment related to DAT in the preclinical stage. In this test, scores of immediate free total recall across the 3 trials and total immediate recall (free and cued) range from 0 to 36 in both cases, whereas scores of delayed free recall and total delayed recall (free and cued) range from 0 to 12.

Language

Boston Naming Test (BNT; Goodglass, Kaplan, Weintraub, & Segal, 2001). For this study we used the 60 item version validated in Spanish young adults in the Neuronorma Project (Peña-Casanova et al., 2009). The test consists of 60 black and white pictures graded in naming difficulty. Each picture is presented individually. The examinee is asked to name each item, and when unable to do so spontaneously, the examiner provides semantic and/or phonemic cues. Correct identification of a picture without cueing or with a semantic cue is awarded one point, whereas errors or identification with phonemic cue earn 0 points. Testing begins with item 1 for all participants and ceases after 4 consecutive errors. No choice recognition task is administered for failed items. Measure of total number of items correctly identified (spontaneously and after semantic cueing) was recorded and considered for the analyses.

Token Test (Renzi and Faglioni, 1978). A shortened version (36 items) was used to evaluate verbal comprehension in this study. Instructions for administration and scoring were taken from the original publication. Test material consists of 20 tokens in two shapes (circles and rectangles), two sizes (big and small), and five colors (red, black, yellow, white, and green). The tokens are laid out according to a fixed configuration in front of the examinee. The test requires the examinee to touch the tokens according to the oral commands provided by the examiner. Thirty-six commands are divided into six stages of increasing complexity. In the first five stages, the examiner repeats the command if the examinee fails to perform the command correctly or does not respond for 5 seconds. A score of 1 point is given when the examinee succeeds in carrying out the command correctly at the first attempt, and 0.5 points are given if he or she succeeds at the second attempt. In the last stage, just one attempt is permitted to carry out the command correctly and a score of 1 or 0 points is given for each item. The task ceases after the examinee fails to correctly carry out five commands in the first stage, or four consecutive commands in any of the remaining stages. Total score in the test was considered for the analyses as a good measure of verbal comprehension.

A.1.2. Functional assessment

The battery included the assessment of functional outcome in daily life in several domains reported to be relevant in DS: adaptive behavior, quality of life, and screening for neuropsychiatric symptoms. Three normalized neuropsychological test were administered to parents: the adult version of the Adaptive Behavior Assessment System (ABAS-II), the parents' version of the Kidscreen-27 for adolescents aged 8 to 18 years, and the Neuropsychiatric Inventory (NPI). In addition, poor sleep quality, as reported by parents, was controlled by means of the Pittsburgh Sleep Quality Index (PSQI) for its possible negative impact on cognitive performance.

Adaptive behavior in daily living

The Adaptive Behavior Assessment System-Second Edition (ABAS-II, adult version; Harrison & Oakland, 2003; Spanish version 2011). ABAS-II was designed, according to AAMS guidelines, for evaluating adaptive skills in people with mental disabilities of a wide age range and across multiple environments. The ABAS-II tool for adults (ages 16 to 89) includes 5 subscales which assess the individual's competence (in terms of behavior frequency) in 10 different skill areas: communication abilities, community use, functional academics, home living, health and safety, leisure, self-care, self-direction, social interaction, and working/labor skills. All answers to this questionnaire were reported by parents. Raw scores of the dimensions and total ABAS score provide a good index of adaptive behavior, higher scores corresponding to greater adaptive skills and independency in everyday living. For the sake of the study, those items rated/reported as guessed by parents were scored as zero in each subscale in order to avoid subjective judgments concerning functional changes. In addition, because most individuals in our sample were unemployed, scores in the work skill area were not included in the analyses and not considered when calculating the total ABAS score.

Quality of life (QoL)

Kidscreen-27 (parents version; Ravens-Sieberer et al., 2007). This instrument assesses quality of life from the child and adolescent's perspective in terms of their physical, mental, and social well-being. The questionnaire measures five dimensions: physical well-being, psychological well-being, autonomy & parents, peers & social support, and school environment, by means of five graded subscales. Only the version for parents was used, and all answers to this tool were self-reported by them. Because a significant proportion of individuals in our sample did not regularly attend school or any educational program, scores in the school dimension were not included in the analyses and were not considered when calculating the total Kidscreen-27 score. Raw scores on the four mentioned dimensions (except school environment) and total score in the scale were included in the study, providing a good measure of quality of life.

Quality of sleep

The Pittsburgh Sleep Quality Index (PSQI; Buysse, III, & Monk, 1989). This questionnaire evaluates the quality and patterns of sleep in older adults. It assesses sleep performance over the previous month across seven different domains: subjective sleep quality, sleep latency, sleep duration, habitual sleep efficiency, sleep disturbances, use of sleep medication, and daytime dysfunction. Parents reported all answers to each of

the seven areas. Scoring is based on a 0 to 3 Likert scale, where 3 represents the negative extreme or sleep dysfunction. A global score under 5 is considered a subjective estimation of good sleep efficiency.

Neuropsychiatric symptoms

The Neuropsychiatric Inventory (NPI; Cummings, 1997). We used the NPI to characterize the neuropsychiatric symptom profiles of our DS participants and to obtain information on the presence of behavioral changes in these individuals along the study. The inventory explores psychopathology in ten behavioral areas (delusions, hallucinations, agitation/aggression, depression/dysphoria, anxiety, euphoria, apathy, disinhibition, irritability/liability, and aberrant motor behavior) and two neurovegetative ones: sleep and appetite/eating disorders. The tool provides measures for the frequency and severity of the changes observed, as well as for emotional/ psychological distress experienced by the caregivers, in all cases rated on a Likert scale. Changes were assessed over the previous 4 weeks, and responses were reported by parents. The NPI total score indicates the presence and frequency of psychiatric symptoms in the ten behavioral areas, whereas the NPI vegetative one only includes some ratings for sleep and appetite disorders, and the NPI distress one estimates caregiver burden. In all cases, higher scores in the mentioned subscales indicate a greater presence of neuropsychiatric symptoms and distress.

A.2 References

Ball, S. L., Holland, A. J., Treppner, P., Watson, P. C., and Huppert, F. A. (2008). Executive dysfunction and its association with personality and behaviour changes in the development of Alzheimer’s disease in adults with Down syndrome and mild to moderate learning disabilities. *Br. J. Clin. Psychol.* 47, 1–29. doi:10.1348/014466507X230967.

Benton, A. L., Hamsher, K., and Sivan, A. (1976). *Multilingual aphasia exam.* , ed. U. of I. Press Iowa City

Buffalo, E. A., Bellgowan, P. S., and Martin, A. (2006). Distinct roles for medial temporal lobe structures in memory for objects and their locations. *Learn. Mem.* 13, 638–643. doi:10.1101/lm.251906.

Buysse, D., III, C. R., and Monk, T. (1989). The Pittsburgh Sleep Quality Index: a new instrument for psychiatric practice and research. *Psychiatry Res.* 28, 193–213.

Canivez, G., Neitzel, R., and Martin, B. (2005). Construct Validity of the Kaufman brief intelligence test, Wechsler intelligence scale for children-and adjustment scales for children and adolescents. *J. Psychoeduc. …* 23, 15–34.

Culbertson, W., and Zillmer, E. (2005). *Tower of London Drexel University: 2nd Edition (TOL DX): Technical manual.* 2nd Editio. New York: Multi-Health Systems Imc.

Cummings, J. (1997). The Neuropsychiatric Inventory Assessing psychopathology in dementia patients. *Neurology* 48, 10–16. doi:10.1212/WNL.48.5_Suppl_6.10S.

Devenny, D. A., Zimmerli, E. J., Kittler, P., and Krinsky-McHale, S. J. (2002). Cued recall in early-stage dementia in adults with Down's syndrome. *J. Intellect. Disabil. Res.* 46, 472–483.

Goldstein, K., and Scheerer, M. (1953). Tests of abstract and concrete behavior. *Contrib. to Med. Psychol.*

Goodglass, H., Kaplan, E., Weintraub, S., and Segal, O. (2001). *Boston naming test.* , ed. L. & Wilkins Philadelphia

Harrison, P., and Oakland, T. (2003). *Adaptive Behavior Assessment System (; ABAS-II).* Western Ps. Los Angeles, CA

Kaufman, A. & K. N. (1990). *K-BIT: Kaufman brief intelligence test.* , ed. I. American Guidance Service Minnesota.

Peña-Casanova, J., Blesa, R., Aguilar, M., Gramunt-Fombuena, N., Gómez-Ansón, B., Oliva, R., Molinuevo, J. L., Robles, A., Barquero, M. S., Antúnez, C., et al. (2009). Spanish Multicenter Normative Studies (NEURONORMA Project): methods and sample characteristics. *Arch. Clin. Neuropsychol.* 24, 307–319. doi:10.1093/arclin/acp027.

Ravens-Sieberer, U., Auquier, P., Erhart, M., Gosch, A., Rajmil, L., Bruil, J., Power, M., Duer, W., Cloetta, B., Czemy, L., et al. (2007). The KIDSCREEN-27 quality of life measure for children and adolescents: psychometric results from a cross-cultural survey in 13 European countries. *Qual. Life Res.* 16, 1347–1356. doi:10.1007/s11136-007-9240-2.

Renzi, E. De, and Faglioni, P. (1978). Normative data and screening power of a shortened version of the Token Test. *Cortex* 14, 41–49.

Robbins, T. W., and Sahakian, B. (1996). Cambridge neuropsychological test automated battery (CANTAB). *CeNeS Limited, Cambridge.* Available at: http://www.cambridgecognition.com/technology.

Strauss, H., and Lewin, I. (1982). An empirical study of the weigl-goldstein-scheerer color-form test according to a developmental frame of reference. *J. Clin. Psychol.* 38, 367–375.

Wechsler, D. (1997). *Wechsler Adult Intelligence Scale-Third Edition.* New York: Psychological Corporation.

Supplementary Table B.1. Sequential order testing and average administration time to complete each of the test.

	Average Administration Time
CANTAB	
MOT: Motor Screening	3 minutes
PRM: Paired Recognition Memory (immediate)	5 minutes
SRT: Simple Reaction Time	6 minutes
SSP: Spatial Spam (direct mode)	5 minutes
SSP: Spatial Spam (revered mode)	5 minutes
PRM: Pattern Recognition Memory (delayed recall)	2 minutes
PAL: Paired Associates Learning	10 minutes
Paper and Pencil Test	
Digit forward and backward spam	5 minutes
Word Fluency	1 minute
Cats and Dogs	2 minutes
Weigl Sort Test	5 minutes
Cued Recall Test	10 minutes
Token Test	10 minutes
TOL-DX	15 minutes
Boston Naming Test	5 minutes

Supplementary Table B.2. Cognitive performance in Down syndrome individuals of verbal episodic memory and executive functions assessed with tests for intellectual disability.

	Down syndrome			
	Mean [A]	***Range (min-max)***	***Age range***	***n***
Executive Functions				
Mental flexibility				
Weigl Sort Test: Total score	*2.2 (1. 7)*	*0 - 5*	*16 – 34*	*85*
Inhibition				
Cats and Dogs: Total time (s)	*25.3 (15.6)*	*10 - 111*	*16 – 34*	*79*
Cats and Dogs: Correct score	*15.2 (1.2)*	*10 - 16*	*16 – 34*	*79*
Verbal Episodic Memory				
CRT: A1-A3 Free immediate recall	*18.9 (6.0)*	*0 - 31*	*16 – 34*	*82*
CRT: A1-A3 Total recall	*33.5 (6.8)*	*0 - 36*	*16 – 34*	*82*
CRT: A1-A3 Repetitions	*2.2(2.9)*	*0 - 13*	*16 – 34*	*82*
CRT: A1-A3 Intrusions	*0.2 (0.7)*	*0 - 4*	*16 – 34*	*82*
CRT: Free delayed recall	*7.1 (2.6)*	*0 - 12*	*16 – 34*	*80*
CRT: Total delayed recall	*11.2 (2.3)*	*0 - 12*	*16 – 34*	*80*
CRT: Delayed recall repetitions	*0.7 (1.5)*	*0 - 8*	*16 – 34*	*80*
CRT: Delayed recall intrusions	*0.2 (0.6)*	*0 - 3*	*16 – 34*	*80*

(A) Results are presented as mean (standard deviation) and expressed in terms of raw scores.

Supplementary Table B.3. Functional performance in Down syndrome individuals of adaptive behavior, quality of life, quality of sleep, and neuropsychiatric symptoms

	Down syndrome			
	Mean(SD) [A]	*Range (max-min)*	*Age range*	*n*
Adaptive Behavior				
ABAS-Communication	*80.3 (12.7)*	*10 - 75*	*16 - 34*	*86*
ABAS-Community Use	*61.5 (13.2)*	*6 - 63*	*16 - 34*	*86*
ABAS-Functional Academics	*67.4 (17.9)*	*5 - 79*	*16 - 34*	*86*
ABAS-Home Living	*64.3 (12.1)*	*4 - 65*	*16 - 34*	*86*
ABAS-Health & Safety	*57.8 (9.5)*	*15 - 60*	*16 - 34*	*86*
ABAS-Leisure	*68.2 (11.5)*	*16 - 69*	*16 - 34*	*86*
ABAS-Self-Care	*87.5 (8.3)*	*37 - 75*	*16 - 34*	*86*
ABAS-Self-Direction	*73.5 (14.7)*	*10 - 75*	*16 - 34*	*86*
ABAS-Social skills	*75.3 (11.6)*	*9 - 75*	*16 - 34*	*86*
ABAS-Work	--	--	--	--
ABAS-Total Score	*635.9 (90.9)*	*220 - 627*	*16 - 34*	*86*
Quality of Life				
Kidscreen 27-Physical	*18.3 (3.6)*	*10-25*	*16 - 34*	*75*
Kidscreen 27-Psychological	*29.7 (3.7)*	*20-35*	*16 - 34*	*75*
Kidscreen 27-Autonomy & Parents	*31.4 (2.8)*	*25-35*	*16 - 34*	*80*
Kidscreen 27-Peers & Social	*15.4 (4.2)*	*4-20*	*16 - 34*	*79*
Kidscreen 27-School	---	---	--	--
Kidscreen 27-Total score	*112.0 (11.9)*	*87-135*	*16 - 34*	*53*
Quality of Sleep				
PSQ-Total score	*2.5 (1.7)*	*0-9*	*16 - 34*	*60*
Neuropsychiatric Sx				
NPI-Total score (dom:1-10) [B]	*0.8 (2.5)*	*0-20*	*16 - 34*	*85*
NPI-Sleep & Appetite disturb.	*0.1 (0.9)*	*0-8*	*16 - 34*	*85*
NPI-Caregiver distress	*0.4(1.5)*	*0-12*	*16 - 34*	*85*

(A) Results are presented as mean (standard deviation) and expressed in terms of raw scores, (B) Results indicate the presence and frequency of psychiatric symptoms on all behavioral areas of the NPI except sleep and appetite disorders.
